# Generalizing Koopman Theory to Allow for Inputs and Control

**DOI:** 10.1137/16M1062296

**Published:** 2018-03-27

**Authors:** Joshua L. Proctory, Steven L. Bruntonz, J. Nathan Kutzx

**Affiliations:** ‡ Department of Mechanical Engineering and Department of Applied Mathematics, University of Washington, Seattle, WA 98195 (sbrunton@uw.edu).; § Department of Applied Mathematics, University of Washington, Seattle, WA 98195 (kutz@uw.edu).

**Keywords:** DMD, Koopman, input-output, DMDc, spatio-temporal, 65P99, 37M99, 37M10, 37N10, 37N35, 37N25

## Abstract

We develop a new generalization of Koopman operator theory that incorporates the e ects of inputs and control. Koopman spectral analysis is a theoretical tool for the analysis of nonlinear dynamical systems. Moreover, Koopman is intimately connected to dynamic mode decomposition (DMD), a method that discovers coherent, spatio-temporal modes from data, connects local-linear analysis to nonlinear operator theory, and importantly creates an equation-free architecture for the study of complex systems. For actuated systems, standard Koopman analysis and DMD are incapable of producing input-output models; moreover, the dynamics and the modes will be corrupted by external forcing. Our new theoretical developments extend Koopman operator theory to allow for systems with nonlinear input-output characteristics. We show how this generalization is rigorously connected to a recent development called dynamic mode decomposition with control. We demonstrate this new theory on nonlinear dynamical systems, including a standard susceptible-infectious-recovered model with relevance to the analysis of infectious disease data with mass vaccination (actuation).

## 1. Introduction

We introduce a new method called Koopman with inputs and control (KIC) that generalizes Koopman spectral theory to allow for the analysis of complex, input-output systems. Koopman operator theory, which is built on the seminal contribution of Bernard Koopman in 1931 [25], is a powerful and increasingly prominent theory that allows one to transform a nonlinear dynamical system into an infinite-dimensional, linear system [25, 31, 42]. Linear operator theory [12], specifically eigenfunction expansion techniques, can then be used to construct solutions of the original system. As such, Koopman theory is perhaps an early theoretical predecessor of what is now called *nonlinear manifold learning*, i.e., discovering nonlinear manifolds on which data live. In Koopman theory, the data is collected from a nonlinear dynamical system. Candidate manifolds are constructed from observables of the original state-space variables. In our KIC innovation, we consider a nonlinear dynamical system with inputs and outputs, thus requiring a generalization of Koopman’s original definition. We demonstrate the method on a number of examples to highlight the e ectiveness and success of the technique. Importantly, the Koopman methodology is data-driven, model-free, and capable of discovering the underlying dynamics and control of a given system from data alone. This makes it an attractive data-driven architecture in modern dynamical systems theory.

Although proposed more than eight decades ago, few results followed the original formulation by Koopman [25]. This was partly due to the fact that there did not exist an e cient method to compute the Koopman operator itself. Additionally, even if an algorithm had been proposed, there were no computers available to compute them in practice during that time period. Interest was revived once again in 2004 by Mezić and Banaszuk [33] and in 2005 by Mezić [31], who showed that Koopman theory could be used for the spectral analysis of nonlinear dynamical systems. Two critical and enabling breakthroughs came shortly after. Schmid and Sessterhen in 2008 [46] and Schmid in 2010 [43] proposed the dynamic mode decomposition (DMD) algorithm for decomposing complex, spatio-temporal data, and in 2009, Rowley et al. [42] showed that the DMD was, in fact, a computation of the Koopman operator for linear observables. Most recently, Tu et al. [48] generalized and improved the DMD algorithm and definition to its current, state-of-the-art form. The combined work of Mezić, Rowley, Schmid, and their coworkers thus laid the theoretical foundations that have led to the tremendous subsequent success of the DMD/Koopman method. In a very short period of time since, DMD theory has been applied with great success to a broad set of domain sciences including complex fluid flows [45, 43, 44, 13, 2, 48, 47], foreground/background separation in video streams [14], epidemiology [37], and neuroscience [3]. The theory also allows for critical enabling theoretical augmentations that can take advantage of compression and sparsity [22, 7, 16], multiresolution/multiscale phenomenon [27], denoising [9, 18], data fusion [52], extended and kernel DMD [51, 50], and control [36]. Indeed, our objective is to describe how Koopman operator theory can be generalized to include the analysis of input-output systems. Further, we demonstrate how Koopman is fundamentally connected to dynamic mode decomposition with control (DMDc), a recently developed extension of DMD for input-output systems [36] which has already been successfully applied to simulation data of a rapidly pitching airfoil [10].

The rapid adoption of Koopman theory across a number of scientific and engineering fields [8, 32] is not surprising. Its fundamental success stems from the fact that it is an *equation-free* method, relying on data alone to reconstruct a linear dynamical system characterizing the underlying nonlinear system. Such linear systems may be characterized using basic methods from ordinary di erential equations and spectral analysis, as shown by Mezić [31]. The method can be applied to high-dimensional measurement data collected from complex systems where governing equations are not readily available. Computing a numerical approximation of the Koopman operator can be orders of magnitude faster than solving for solutions of partial di erential equations with complex domains. This Koopman mode decomposition (KMD) utilizes the numerically efficient DMD algorithm. However, KMD typically includes a set of judiciously chosen observables, whereas DMD relies solely on linear observables. The set of observables for KMD is often larger and includes nonlinear functions. KMD utilizes these observables to find a coordinate system that allows for a linear operator to describe the dynamics of these observables. In this manuscript, we show how KIC inherits the advantageous characteristics of Koopman theory, while also extending the domain of applicability to input-output systems.

The control of high-dimensional, nonlinear systems is a challenging task that is of paramount importance for applications such as flow control [5] and eradicating infectious diseases [37]. The construction of e ective controllers typically relies on relatively few states, a computationally feasible model to implement, and fast solvers to minimize latencies introduced by computing estimates of the system [1]. Further, control laws often rely on solving a single large Riccati equation (*𝓗*_2_) or iteratively through sets of equations (*𝓗_∞_*). For modern engineering systems with high-dimensional measurement data and possibly high-dimensional input data, the requirements of the controllers are too restrictive. Thus, most practical methods for handling these modern systems rely heavily on dimensionality-reduction techniques. These *model reduction* techniques typically employ the singular value decomposition to discover low-dimensional subspaces where the dynamics evolve [19]. On these low-dimensional subspaces, controllers can be described, constructed, and implemented [35, 23, 19, 41, 40, 39, 53, 17]. This paradigm is exemplified in the classic method called balanced truncation, which utilizes both the low-dimensional controllable *and* observable subspaces to produce a balanced, reduced-order model for control [35]. Notably, balanced truncation has been extended and generalized to handle high-dimensional measurement data by a method called balanced proper orthogonal decomposition (BPOD). The method, however, requires a linear adjoint calculation [28, 49, 40, 20], which is not possible in typical data-driven experiments.

The models produced by BPOD have been shown to be equivalent to the input-output models produced by the eigensystem realization algorithm (ERA), a method developed to be used on linear and low-dimensional systems [29]. ERA and the observer Kalman identification method (OKID) are a part of a class of methods developed for system identification [23, 24, 11]. Similar to DMD and DMDc, system identification methods are inherently equation-free, acting only on measurement and input data. In fact, DMD has been shown to be intimately connected to ERA and OKID as well as other subspace identification methods such as the numerical algorithms for subspace state space system identification (N4SID) [38, 48, 36]. In this manuscript, we will establish the connection between KIC and DMDc for linear input-output systems. KIC can be interpreted as a general framework for nonlinear system identification.

The outline of the paper is as follows. Section 2 describes the background on Koopman operator theory and its connections to DMD. Section 3 describes the new development KIC and the strong connections to DMDc. Section 4 presents a number of numerical examples including nonlinear input-output systems.

## 2. Background: Koopman and dynamic mode decomposition

Koopman operator theory and DMD are powerful and intimately connected methods for analyzing complex systems. Data collected from numerical simulations, experiments, or historical records can be analyzed by Koopman and DMD. These methodologies can identify important dynamic characteristics relevant for prediction, bifurcation analysis, and parameter optimization. This section provides the mathematical background for Koopman operator theory and DMD [31, 46, 45, 42, 48]. Further, the section includes a description of DMD with control, allowing the DMD framework to be applied to systems with exogenous forcing.

### 2.1. The Koopman operator for dynamical systems

The *Koopman operator* can transform the analysis of a finite-dimensional, nonlinear dynamical system into the analysis of an infinite-dimensional, linear system [25]. Standard spectral analysis of this linear Koopman operator is a powerful methodology to investigate flows arising from nonlinear dynamical systems [31, 42, 8, 32]. In this section, we describe Koopman operator theory.

Consider the discrete nonlinear dynamical system,

(2.1) $${\text{x}}_{k+1}=\text{f}({\text{x}}_{k}),$$

evolving on a smooth manifold *𝓜* where x*_k_*∊ 𝓜. The function is a map from *𝓜* to itself, and *k* is an integer index. We could equivalently describe Koopman operator theory for continuous-time systems, but here we focus on the discrete-time setting reflective of most engineering applications. We define a set of scalar valued functions *g* : *𝓜* → ℝ, which are called observables. The set of observables defines an infinite-dimensional Hilbert space *𝓗* . For example, this space could consist of the Lebesque square-integrable functions. Previous investigations have focused on analyzing the appropriate spaces on which the Koopman operator acts [34] and global stability properties [30]. In this article, we will use polynomial functions as observables, which are square-integrable assuming they are defined on a compact set. The Koopman operator *𝓚* acts on this set of observables:

(2.2) $$Kg\left(x_{k}\right)≜g\left(f\left(x_{k}\right)\right)$$

The Koopman operator is linear and *infinite* -dimensional. The nonlinear dynamical system is often considered finite-dimensional but can be infinite-dimensional. The linear characteristics of the Koopman operator allow us to perform an eigendecomposition of *𝓚*:

(2.3) $$K\varphi_{j}(x)=\lambda_{j}\varphi_{j}(x),\;j=1,2,\ldots ,\infty$$

Consider a vector-valued observable $𝓜\to {\mathbb{R}}^{n_{y}}$. Using the infinite expansion shown in (2.3), the observable g can be rewritten,

(2.4) $$g(x)=\left[\begin{array}{c} g_{1}(x)\\ g_{2}(x)\\ g_{3}(x)\\ \vdots \\ g_{n_{y}}(x) \end{array}\right]=\sum_{j=1}^{\infty }\;\varphi_{j}(x)v_{j}$$

as long as the *n_y_* components of g lie within the span of eigenfunctions *ϕ_j_* . The vector-valued coe cients v*_j_* are called Koopman modes. Measure-preserving flows, as those originally considered in [25], allow for a specific description of the Koopman modes based on projections of the observables:

(2.5) $$g(x)=\sum_{j=1}^{\infty }\;\varphi_{j}(x)\left[\begin{array}{c} <\varphi_{j},g_{1}>\\ <\varphi_{j},g_{2}>\\ <\varphi_{j},g_{3}>\\ \vdots \\ <\varphi_{j},g_{n_{y}}> \end{array}\right]=\sum_{j=1}^{\infty }\;\varphi_{j}(x)v_{j}$$

where the inner product is with respect to *ℋ* . The Koopman operator *𝓀* is defined for all observables. We later denote a finite-dimensional approximation of the Koopman operator (from data) as **K**. Rearranging terms from (2.2) and (2.3) provides a new representation of the observable g in terms of Koopman modes and the corresponding Koopman eigenvalues λ*_j_*:

(2.6) $$Kg\left(x_{k}\right)=g\left(f\left(x_{k}\right)\right)=\sum_{j=1}^{\infty }\;\lambda_{j}\varphi_{j}\left(x_{k}\right)v_{j}$$

where the Koopman eigenvalues λ*_j_* describe the growth/decay and oscillatory frequency for each Koopman mode,v*_j_*. For DMD, *ϕ_j_* (**x**) is a constant and is typically absorbed into each of the modes. The eigenfunctions *ϕ* (**x**) are the inner product of the state x of the linear Koopman operator w*_j_* when the underlying system is linear and the observables are the identify, i.e., **g**(**x**) = x [42].

A significant amount of recent work has focused on identifying a procedure for constructing a set of observables g that will uncover an approximate Koopman operator **K** [50, 4]. For example, one procedure augments the measured state with a set of nonlinear functions, e.g., x^2^, x^3^, sin(**x**).

### 2.2. Koopman and DMD

In this background section, we show how Koopman operator theory is connected to DMD. Given a set of internal states x*_k_*, where *𝓚* = 1, 2*, . . . , m*, the measurements of the system states can be described by the following:

(2.7) $$y_{k}=g\left(x_{k}\right),\;y_{k+1}=z_{k}=g\left(f\left(x_{k}\right)\right)$$

Each of the measurements can be collected to form two large data matrices:

(2.8) $$Y=\left[\begin{array}{cccc} | & | & | & \;\\ y_{1} & y_{2} & \ldots & y_{m}\\ | & | & | & \; \end{array}\right],\;Z=\left[\begin{array}{cccc} | & | & & |\\ z_{1} & z_{2} & \ldots & z_{m}\\ | & | & & | \end{array}\right]$$

These snapshot matrices can be used to define the DMD.

Definition 2.1 (dynamic mode decomposition (Tu et al. [48])).

*The DMD of the measurement pair* (Y,**Z**) *is given by the eigendecomposition of*A*, where* **A** ≜ **ZY** *^†^* *and†* *is the pseudoinverse.*

The measurements of the state, x_1_, x_2_, . . . , x*_m_*, do not have to be sequentially sampled. The important relationship is between the current and future measurements, y*_k_* and z*_k_*, respectively. The states x*_i_* do not have to be collected from a single trajectory of **f**. Of course, in collecting data from an experiment or historical records, the data is typically collected along a single trajectory in phase space.

DMD can be computed from the measurement pair (**Y, Z**) by finding eigenvectors and eigenvalues that satisfy the standard eigenvalue problem:

(2.9) $$Av_{j}=\lambda_{j}v_{j}$$

Assuming the matrix A has a full set of eigenvectors, each measurement column y*_k_* can be represented by the eigenvectors of **A**:

(2.10) $$g\left(x_{k}\right)=\sum_{j=1}^{n}\;c_{jk}v_{j}$$

If we have linearly consistent data, the relationship **Ay*_k_* = z*_k_*** is satisfied allowing us to apply the operator A to (2.10):

(2.11a) $$\begin{array}{r} g\left(f\left(x_{k}\right)\right)=z_{k}=A\sum_{j=1}^{n}\;c_{jk}v_{j} \end{array}$$

(2.11b) $$=\sum_{j=1}^{n}\;Ac_{jk}v_{j}$$

(2.11c) $$=\sum_{j=1}^{n}\;\lambda_{j}c_{jk}v_{j}$$

In the case of linearly consistent data matrices, the DMD modes and eigenvalues of (2.11) correspond to the Koopman modes of (2.6). The data matrices **Y** and **Z** are linearly consistent if and only if the null space of **Z** is contained in the null space of **Y**. We refer the reader to [48] for a more detailed description of linearly consistent data.

### 2.3. Dynamic mode decomposition with control

DMDc extends DMD to handle complex systems with inputs and control [36]. The DMD eigenvalues and dynamic modes may be corrupted without including information from exogenous forcing or controller inputs. The time-varying inputs to the system can be collected to form another data matrix similar to those found in (2.8):

(2.12) $$Y_{u}=\left[\begin{array}{cccc} | & | & & |\\ u_{1} & u_{2} & \ldots & u_{m}\\ | & | & & | \end{array}\right]$$

DMDc combines the input snapshot matrix Y*_u_* with the state snapshot matrices (Y,**Z**) to disambiguate the state dynamics from the impact of the inputs. DMDc is defined as follows.

Definition 2.2 (dynamic mode decomposition with control (Proctor, Brunton, and Kutz [36])).

*The DMDc of the measurement trio* (**Y, Z, Y***_u_*) *is given by the eigendecomposition of the operator* A *where* $\tilde{G}=\left[A\;B\right]\;and\;\tilde{G}≜Z{\left[\overset{Y}{Y_{u}}\right]}^{†}={\mathrm{Z\Omega}}^{†}$.

DMDc utilizes three measurement matrices (**Y, Z, Y***_u_*) producing a nonsquare operator $\tilde{G}$ that helps identify input-output characteristics. Typically, the measurement matrices are constructed with full-state access and full-input access such that **Y** = [x_1_ x _2_*…* x*_m_*], Y*_u_* = [u_1_ u_2_ *…*u *_m_*], **Z** = [x_2_ x_3_ *…* x*_m_*_+1_]. The DMD modes from the measurement trio can be found by performing the singular value decomposition:

(2.13) $$\tilde{G}v_{j}=\sigma_{j}q_{j}$$

Assuming the matrix $\tilde{G}$ has a full set of singular vectors, each measurement column of Ω can be represented by expanding with the eigenvectors v*_j_*:

(2.14) $$\left[\begin{array}{l} y_{k}\\ u_{k} \end{array}\right]=\sum_{j=1}^{n}\;\varphi_{j}v_{j}$$

If we have linearly consistent data, the relationship $\tilde{G}\left[\begin{array}{c} y_{k}\\ u_{k} \end{array}\right]$ is satisfied allowing us to apply the operator $\tilde{G}$ to (2.10) giving

(2.15a) $$\begin{array}{rr} z_{k}=\tilde{G}\sum_{j=1}^{n}\;c_{jk}v_{j} & \end{array}$$

(2.15b) $$=\sum_{j=1}^{n}\;\sigma_{j}c_{jk}q_{j}$$

The DMDc identification of operators **A** and **B** can be juxtaposed with a standard model-based approach from linear system theory. The standard linear control model is given by the following:

(2.16a) $$x_{k+1}={\mathrm{Ax}}_{k}+{\mathrm{Bu}}_{k}$$

(2.16b) $$y_{k}={Cx}_{k}+Du_{k}$$

Clearly, DMDc and standard linear models are fundamentally connected [36]. Similarly, section 2.2 illustrates the link between DMD and Koopman operator theory. These connections help frame the motivation around generalizing Koopman operator theory with inputs and control. In this article, we develop KIC as a methodology for analyzing complex systems with nonlinear input-output characteristics. Further, we will show that, similar to Koopman theory, this methodology is completely data-driven and related to DMDc.

## 3. Generalizing Koopman to allow for inputs and control

In this section, Koopman operator theory is generalized to include exogenous inputs and control. We then demonstrate how the new theoretical framework connects to linear systems theory. Further, we describe the connection of KIC to DMDc.

### 3.1. Koopman with inputs and control

**Figure 1.**
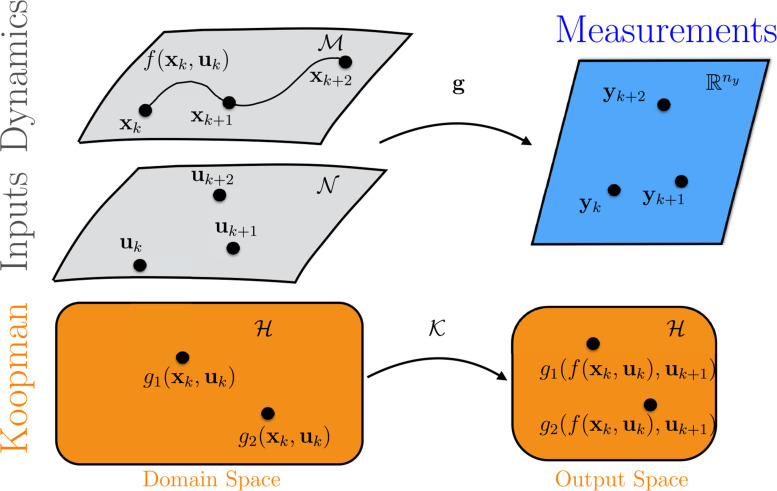
This figure illustrates the Koopman operator with inputs and control. The internal dynamics and the inputs are measured with the observable *y_k_* = *g*(x*_k_*, u*_k_*) . The bottom row indicates the goal of the Koopman operator with inputs and control: to find an operator that propagates all observables *g* *_j_* (x*_k_*,u*_k_*) to the same observable in the future *g* *_j_* ( *f* (x*_k_*,u*_k_*) , u*_k_*_+1_).

Consider a nonlinear dynamical system that allows for external inputs

(3.1) $$x_{k+1}=f\left(x_{k},u_{k}\right)$$

where x ∊ 𝓜, u ∊ *N*, and *𝓜* and *𝒩* are both smooth manifolds. As before, we dispense with the manifolds and consider x *2* ℝ*^n^* *^x^* and u *2* ℝ*^n^* *^u^*. Further, we do not require **u** to be constrained to a manifold. We define a set of scalar-valued observables that are functions of the state *and* the input where *g* : *𝓜 × 𝒩 ℝ*. Each observable is an element of an infinite-dimensional Hilbert space *𝓗*. Figure 1 illustrates the relationship between the underlying dynamics, inputs, and measurements and the Koopman operator. The Hilbert space can be defined by the Lebesque square-integrable functions, other appropriate spaces [34], or polynomial functions defined on a compact set. Note that *ℋ* contains observables that depend on the state, e.g., *g* (x, **u**) = *x*_1_, the inputs, i.e., *g* (x, **u**) = *u*_1_, and mixed terms, i.e., *g* (x, **u**) = *x*_1_*u*_1_. These observables can be combined into a vector-valued observable **g**(x, **u**) to compute a finite-approximation of the Koopman operator.

The KIC *𝒦* : *ℋ → ℋ* acts on the Hilbert space of observables

(3.2) $$Kg\left(x_{k},u_{k}\right)≜g\left(f\left(x_{k},u_{k}\right),u_{k+1}\right)$$

The KIC definition can be modified depending on the type of input. Consider the following input types:

1. *Closed-loop control*: The input is generated from a state-dependent controller, u*_k_* = h (x*_k_*). The KIC operator can be defined for a closed-loop controller by the following:
  𝒦*g* (x*_k_*, u*_k_*) ≜ *g* (**f**(x*_k_*, u*_k_*), **h**(**f**(x*_k_*, u*_k_*))). In this case, the KIC operator can be rewritten using (3.1) in terms of only the state: *Kg* (x*_k_*, **h**(x*_k_*)) = *g* (x*_k_*_+1_, **h**(x*_k_*_+1_)). Thus, the KIC operator for a given closed-loop control law reduces to the Koopman operator for the associated autonomous system.
2. *Open-loop inputs*:
  - The input is generated from a constant forcing term. The KIC operator can be defined for a constant input **c** by the following: *𝒦g* (x*_k_*, **c**) ≜ *g* (**f**(x*_k_*, **c**), **c**). For each input **c**, the KIC operator can be reduced to the Koopman operator for the associated autonomous system, inheriting the spectral properties and characteristics. However, the KIC operator now represents a family of autonomous Koopman operators defined at constant values of input. (b) The input is generated from an exogenous forcing term, such as a time-varying input or random disturbance. In this case, the KIC operator can be defined by the following: *Kg* (x*_k_*, u*_k_*) ≜ *g* (**f**(x*_k_*, u*_k_*), u*_k_*_+1_). Note that this definition suggests that the KIC operator can propagate both the state dynamics and unknown inputs. This case has also been investigated with the shift operator of a known input profile [26].
  - An open-loop input can also come in the form of a process with its own internal dynamics, u*_k_*_+1_ = f*_u_* (u*_k_*). The KIC operator can be defined by the following: *Kg* (x*_k_*, u*_k_*) ≜ *g* (**f**(x*_k_*, u*_k_*), f*_u_* (u*_k_*)). As in the case of closed-loop control, the KIC operator can be reduced to the autonomous Koopman operator by treating the inputs as states. The KIC operator can be used to investigate independent dynamic processes acting on each other, such as those found in dynamic networks and multiscale systems.

**Figure 2.**
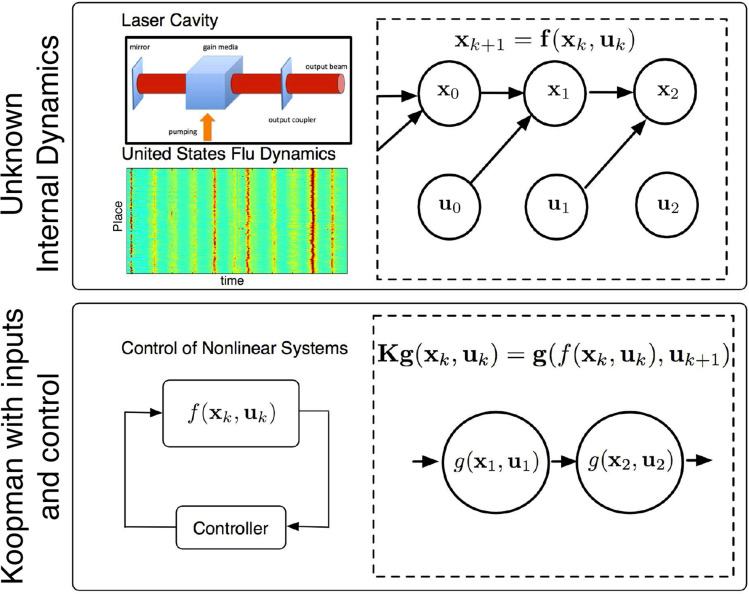
An illustration motivating the development of Koopman operator theory with inputs and control. The first row shows a complex system evolving in time with unknown, underlying dynamics. Both systems, an optical cavity and epidemiological system, can have exogenous forcing. The second row shows the goal of Koopman operator theory: to discover an operator that can propagate forward in time a set of measurements for prediction and control without an explicit model.

In this novel definition of the Koopman operator, the primary goal is to identify a *𝒦* that represents both the evolution of the nonlinear dynamics and the impact of an arbitrary control signal on the system. Figure 2 illustrates this general motivation for KIC. In practice, the recovery of a finite-approximation of *𝒦* for any arbitrary input signal will require a rich set of measurements, control signal profiles, and initial conditions.

It’s important to note that the analysis of *𝒦* can reduce to Koopman operator theory for an associated autonomous dynamical system, illustrated in the case for closed-loop control 1 and open-loop control 2(a) and 2(c). For systems with closed-loop control, vector-valued observables, *𝓚***g**(x*_k_*, u*_k_*) = **g**(x*_k_*_+1_, **h**(x*_k_*_+1_)), can be reduced to (2.4). For example, in the case where observables are linear functions of the state and input, **u** can be adjoined to the state **x** constructing an augmented state variable $\left[x^{T}\;u^{T}\right]≜g\left(f\left(x_{k},\;u_{k}\right),\;u_{k+1}\right).$. Koopman operator theory can be applied where observables are functions of this new augmented state; thus KIC inherits the spectral properties and characteristics from Koopman operator theory for autonomous systems. However, carefully partitioning the observables that depend on the state from those that depend on the inputs can help disambiguate the state dynamics from the e ects of inputs. Further, the clear algorithmic connection to DMD o ers a procedure to treat data collected from multiple state trajectories, initial conditions, and control signals; see section 3.3 for how to construct the KIC data matrices. This observation will be utilized in connecting KIC to DMDc in section 3.3.

The augmentation procedure can be used for open-loop control where the inputs have their own internal dynamics. The Koopman operator will be able to discover both the input and state dynamics. As a motivating example, the Koopman operator could be applied to systems with multiscale dynamics allowing for the identification of microscale behavior driving macro-scale outputs, such as those found in climate or epidemiological systems. DMD, described in section 2.2, has been previously extended to handle data from multiscale processes [27]. Similarly, a common method for analyzing nonautonomous dynamical systems is to treat time as a state, creating another augmented state. Then, the vector field *f* is augmented with a simple ODE, $\dot{t}=1$ [15].

In contrast, open-loop control without dynamics, such as those found in standard system identification procedures, does not require a KIC operator that can predict the future inputs. The KIC operator can be defined such that future inputs are known a priori. This has also been proposed in [26], where a shift operator is utilized to step the input forward in time. The KIC operator can also be defined such that *𝒦g* (x*_k_*, u*_k_*) ≜ *g* (**f**(x*_k_*, u*_k_*), 0) imposing a realistic constraint on future input prediction. Practically, this assumption amounts to utilizing observables of the current state and inputs to propagate forward *only* the observables that depend solely on the state. This perspective helps fundamentally link KIC to DMDc and system identification methods.

Generally, the linear characteristics of the KIC allow us to perform an eigendecomposition of *𝒦* given in the standard form:

(3.3) $$K\varphi_{j}(x,u)=\lambda_{j}\varphi_{j}(x,u),\;j=1,2,\ldots \infty$$

The operator is spanned by eigenfunctions that are defined by the inputs and state. Using the infinite expansion shown in (3.3), the vector-valued observable **g** can be rewritten in terms of the right eigenfunctions *ϕ_j_*,

(3.4) $$g(x,u)=\left[\begin{array}{c} g_{1}(x,u)\\ g_{2}(x,u)\\ \vdots \\ g_{n_{y}}(x,u) \end{array}\right]=\sum_{j=1}^{\infty }\;\varphi_{j}(x,u)v_{j}$$

where *n_y_* is the number of observables. The Koopman with inputs and control operator can be applied to these observables,

(3.5) $$Kg\left(x_{k},u_{k}\right)=g\left(f\left(x_{k},u_{k}\right),u_{k+1}\right)=\sum_{j=1}^{\infty }\;\lambda_{j}\varphi_{j}\left(x_{k},u_{k}\right)v_{j}$$

Note that the expansion is in terms of Koopman eigenfunctions with vector-valued coe cients that we call Koopman modes v*_j_* . Koopman operator theory now allows for observables that are functions that depend on both the state and inputs.

### 3.2. KIC for linear systems

In this subsection, we demonstrate how KIC can be applied to linear systems. Consider the linear dynamical system with inputs

(3.6) $$x_{k+1}=Ax_{k}+Bu_{k}$$

We consider full-state access and full-input access by choosing observables that are the identity, i.e., **g**(x, **u**) = [x*^T^*u*^T^*]*^T^*. The linear dynamical system can be rewritten in terms of **y**:

(3.7a) $$\left[\begin{array}{c} x_{k+1}\\ u_{k+1} \end{array}\right]=\left[\begin{array}{ll} G_{11} & G_{12}\\ G_{21} & G_{22} \end{array}\right]\left[\begin{array}{c} x_{k}\\ u_{k} \end{array}\right]$$

(3.7b) $$y_{k+1}={\mathrm{Gy}}_{k}$$

The eigenvalues of **G** are also the eigenvalues of *𝒦* and the left and right eigenvectors of **G** are related to the eigenfunctions of *𝒦* . The description of this linear system for an input-output system is clearly not standard. Typically, the future state would not include the future input. Later in this section, we discuss how the Koopman operator with inputs and control can be modified to reflect a more standard view of input-output systems, connecting previous work on DMDc [36]. The decomposition of **G**, with eigenvalues λ*_j_* and eigenvectors v*_j_*, is

(3.8) $$Gv_{j}=\lambda_{j}v_{j},\;j=1,2,\ldots ,n$$

The observables can be represented by an expansion in terms of the **v**:

(3.9) $$y=\sum_{j=1}^{n}\;\varphi_{j}(x,u)v_{j}=\sum_{j=1}^{n}\;<z,w_{j}>v_{j}$$

where the inner product is defined with respect to *ℋ*. Also, w*_j_* are the left eigenvectors of the operator **G**. Further, the eigenfunctions *"_j_* are projections of the state on the eigenvectors **w**. For linear systems, the Koopman operator is equivalent to the linear map **G**. Further, the Koopman modes (both the left and right) coincide with the eigenvectors of **G**.

### 3.3. KIC and connections to DMDc

KIC for linear systems with control does not identify input-output systems in a standard form; see section 3.2. However, the KIC framework is highly flexible. KIC is related to a recently developed method called DMDc, described in section 2.3, which does produce standard input-output models. This connection parallels the link between Koopman operator theory and DMD, described in section 2.2 [42].

Similar to section 2.3, we define a set of internal states x*_k_* and internal inputs u*_k_*, where *𝓚* = 1, 2*, . . . , m* . The observables **g**(x*_k_*, u*_k_*) can be partitioned into those dependent on the state y*_x_*, control y*_u_*, and both y*_xu_*:

(3.10) $$y_{k}=\left[\begin{array}{c} y_{x,k}\\ y_{xu,k}\\ y_{u,k} \end{array}\right]=g\left(x_{k},u_{k}\right),\;z_{k}=\left[\begin{array}{c} z_{x,k}\\ z_{xu,k}\\ z_{u,k} \end{array}\right]=g\left(f\left(x_{k},u_{k}\right),u_{k+1}\right)$$

As with DMDc, the set of states x*_k_* and inputs u*_k_* does not need to be collected from a single trajectory of the dynamical system [48]. Each of the measurements can be collected to form two large data matrices:

(3.11) $$y_{k}=\left[\begin{array}{c} y_{x}\\ y_{\mathrm{xu}}\\ y_{u} \end{array}\right]=\left[\begin{array}{cccc} | & | & & |\\ y_{x,1} & y_{x,2} & ... & y_{x,m}\\ | & | & & |\\ | & | & & |\\ y_{xu,1} & y_{xu,2} & ... & y_{xu,m}\\ | & | & | & |\\ | & | & | & |\\ y_{u,1} & y_{u,2} & ... & y_{u,m}\\ | & | & & | \end{array}\right],\;z_{k}=\left[\begin{array}{c} z_{x}\\ z_{xu}\\ z_{u} \end{array}\right]=\left[\begin{array}{cccc} | & | & & |\\ z_{x,1} & z_{x,2} & ... & z_{x,m}\\ | & | & & |\\ | & | & & |\\ z_{xu,1} & z_{xu,2} & ... & z_{xu,m}\\ | & | & | & |\\ | & | & | & |\\ z_{u,1} & z_{u,2} & ... & z_{u,m}\\ | & | & & | \end{array}\right]$$

The connection to DMDc can be established by choosing linear observables for the state and control, such that **Y***_x_* = [x_1_ x_2_ *…*x*_m_*], **Y***_u_* = [u_1_ u_2_ *…*u*_m_*], **Z***_x_* = [x_2_ x_3_ *…* x*_m_*_+1_], and **Y***_xu_* = **Z***_xu_* = **Z***_u_* = 0. With these linear observables, the KIC operator reduces to DMDc.

A judicious choice of observables **g**(x*_k_*, u*_k_*) can transform the state and input measurements into a coordinate system that allows for a linear operator to represent the nonlinear input-output dynamics. DMDc, however, utilizes linear observables with full state and input access. DMDc and KIC are similar, though, in that the KIC operator can be numerically approximated through the same e cient numerical algorithm as DMD. Further, the flexible KIC framework allows for the construction of a nonlinear input-output operator when **Z***_xu_* = **Z***_u_* = **0**.

### 3.4. Adapting KIC to allow for di erent domain and output spaces

In this subsection, we discuss the flexibility of the KIC architecture to allow for di erent domain and output spaces. Specifically, we illustrate how the Koopman operator can be viewed as projecting from the complete Hilbert space *ℋ* to a subspace of *ℋ* . This facilitates a stronger connection to DMDc, as well as recent developments such as the sparse identification of nonlinear dynamics (SINDy) [6].

#### 3.4.1. The inputs are not dynamically evolving

The Koopman operator in (3.2), without dynamically evolving inputs, can be defined by the following: *Kg* (x*_k_*, u*_k_*) ≜ *g* (**f**(x*_k_*, u*_k_*), 0). In this case, the operator is no longer attempting to fit a future input prediction. Instead, this modified Koopman operator propagates the future observables dependent solely on the state. The approximation of the operator becomes

(3.12) $$\left[\begin{array}{c} y_{x,k+1}\\ y_{xu,k+1}\\ y_{u,k+1} \end{array}\right]=\left[\begin{array}{ccc} G_{11} & G_{12} & G_{13}\\ 0 & 0 & 0\\ 0 & 0 & 0 \end{array}\right]\left[\begin{array}{c} y_{x,k}\\ y_{xu,k}\\ y_{u,k} \end{array}\right]$$

which can be reduced to

(3.13) $$\left[\begin{array}{l} y_{x,k+1} \end{array}\right]=\left[\begin{array}{l} G_{11}\;G_{12}\;G_{13} \end{array}\right]\left[\begin{array}{c} y_{x,k}\\ y_{xu,k}\\ y_{u,k} \end{array}\right]$$

KIC connects the nonstandard form found in section 3.2 with system identification methods [36]. Further, the approximate, finite-dimensional Koopman operator **K** can be constructed from the reduced set of data matrices:

(3.14) $$Z_{x}=K\left[\begin{array}{l} Y_{x}\\ Y_{xu}\\ Y_{u} \end{array}\right]$$

This construction of the KIC operator requires a closer inspection of the eigenfunction expansion in (3.3). There is no longer a requirement for having equivalent eigenfunctions *ϕ_j_* for both the domain and output spaces of the operator *𝒦*. Here, the eigenfunctions *ϕ_j_* could be mapped to a restricted subspace of *𝓗* that only concerns the prediction of the future state.

#### 3.4.2. Domain and output spaces for the Koopman operator

We investigate how the output space of the Koopman operator can be restricted to a subspace of *ℋ* . In section 3.1, we illustrated how the Koopman operator is defined on *ℋ* for all observables *g* (x, **u**). This space *ℋ* can be partitioned into subspaces. We illustrate how these subspaces can be utilized to describe the output space of the Koopman operator. We could expand the domain and output spaces of *𝒦* by the following:

(3.15) $$K\varphi_{j}(x,u)=\sigma_{j}\psi_{j}(x,u),\;j=1,2,\ldots \infty$$

where ψ*_j_* are eigenfunctions that span a subspace of *ℋ* . The span of *ϕ* includes the span of ψ, as well as the span of the remaining nonlinear observables on **x** and **u**. The vector of observables **g**(x, **u**) can still be defined as in (3.4). The Koopman operator applied to **g**(x, **u**) becomes

(3.16) $$Kg(x,u)=\sum_{j=1}^{n}\;K\varphi_{j}(x,u)\left[\begin{array}{c} q_{1}\\ q_{2}\\ \vdots \\ q_{n_{z}}\\ 0\\ \vdots \\ 0 \end{array}\right]\approx \sum_{j=1}^{\infty }\;\sigma_{j}\psi_{j}(x)q_{j}$$

where *n_x_* is a smaller set of observables than *n_y_* and q*_j_* are left Koopman modes. This allows for di erent domain and output spaces for the Koopman operator expansion. The distinction provides the practitioner the ability to investigate how the Koopman operator projects from a large domain space of observables that includes linear, nonlinear, and mixed terms to a restricted output space dependent only on the state. This framework allows for a larger number of observables than DMDc, providing a principled method for expanding the domain space of observables. The domain space can include a large functional library expanding the measurement set as in the work by Williams, Kevrekidis, and Rowley [50].

In this framework, the domain space of the Koopman operator can be expanded in linear measurements of the state and input, nonlinear measurements of the state and inputs, and mixed state-input terms. The output space, though, can be restricted to a set that spans, for example, the linear state measurements.

#### 3.4.3. Domain/output spaces of the Koopman operator for linear systems

In this subsection, we discuss how the Koopman operator maps to observables that are of the form *g* (x, **u**) = *g* (**x**). For observables that are the identity on the state and input measurements, the domain space can be represented by a similar expansion of section 3.1, in terms of the right Koopman modes v*_j_* :

(3.17) $$\left[\begin{array}{l} x\\ u \end{array}\right]=y=\sum_{j=1}^{n}\;\varphi_{j}(y)v_{j}=\sum_{j=1}^{n}\;<y,v_{j}>v_{j}$$

The KIC operator *𝒦* can be applied to **y**,

(3.18a) $$Ky=\sum_{j=1}^{n}\;<Ky,q_{j}>q_{j}$$

(3.18b) $$=\sum_{j=1}^{n}\;<y,K^{*}q_{j}>q_{j}$$

(3.18c) $$=\sum_{j=1}^{n}\;<y,\sigma_{j}^{*}v_{j}>q_{j}$$

(3.18d) $$=\sum_{j=1}^{n}\;\sigma_{j}<y,v_{j}>q_{j}$$

where now the output space is expanded by q*_j_*.

## 4. Applications

This section explores the theoretical development of KIC on various linear and nonlinear examples. For examples 1–3, we assume the perspective of the applied scientist where a finite set of measurements exist. The first example explores the implementation of KIC for inputs that are random disturbances, from a controller, or from an external process with dynamics. The second example explores a nonlinear dynamical system with a quadratic nonlinearity well-studied in the Koopman and DMD literature. The final example looks at a canonical susceptible-infected-recovered (SIR) model arising out of the study of infectious diseases. This example illustrates the challenge facing the community applying Koopman and DMD for realistic nonlinear problems.

### 4.1. Example 1—Linear system with inputs

Consider the following linear dynamical system:

(4.1) $${\left[\begin{array}{l} x_{1}\\ x_{2} \end{array}\right]}_{k+1}={\left[\begin{array}{c} \mu x_{1}\\ \lambda x_{2}+\delta u \end{array}\right]}_{k}$$

A similar example can be found in [36]. If *|λ|* and/or *|μ|* is *>* 1, the system is unstable. The goal is to recover the underlying dynamics and input matrix when there are various types of inputs including random disturbances, a state-feedback controller, or a multiscale system. We assume full access to the state and inputs giving the following relationship between the states, inputs, and measurements:

(4.2) $$\left[\begin{array}{l} y_{1}\\ y_{2}\\ y_{3} \end{array}\right]=\left[\begin{array}{c} x_{1}\\ x_{2}\\ u \end{array}\right],\;{\left[\begin{array}{l} y_{1}\\ y_{2} \end{array}\right]}_{k+1}=\left[\begin{array}{cc} \mu & 0\\ 0 & \lambda \end{array}\right]{\left[\begin{array}{l} y_{1}\\ y_{2} \end{array}\right]}_{k}+\left[\begin{array}{l} 0\\ \delta \end{array}\right]y_{3,k}$$

The dynamical system can be rewritten in the KIC form:

(4.3) $${\left[\begin{array}{l} y_{1}\\ y_{2}\\ y_{3} \end{array}\right]}_{k+1}=\left[\begin{array}{l} \mu \;0\;0\\ 0\;\lambda \;\delta \\ a\;b\;c \end{array}\right]{\left[\begin{array}{l} y_{1}\\ y_{2}\\ y_{3} \end{array}\right]}_{k}$$

where *a*, *b*, and *c* depend on the types of inputs. We first investigate when the inputs are random disturbances. Note that we do not expect to recover the coe cients (*a, b, c*) from the random disturbances. Further, the formulation of this problem could have utilized u*_k_*_+1_ = 0 or the shift operator from [26]. Instead, we have chosen u*_k_*_+1_ to illustrate the impact of di erent assumptions on the finite-approximation of the KIC operator.

We collect measurements of the state and inputs to investigate the reconstruction of a finite-dimensional Koopman operator. The following is the first five snapshots of a single realization:

(4.4a) $$Y=\left[\begin{array}{ccccc} 5 & 0.5 & 0.05 & 0.005 & 0.0005\\ 2 & 2.999 & 4.497 & 6.749 & 10.132\\ -0.001 & -0.001 & 0.002 & 0.009 & 0.004 \end{array}\right]$$

(4.4b) $$Z=\left[\begin{array}{ccccc} 0.5 & 0.05 & 0.005 & 0.0005 & 0.00005\\ 2.999 & 4.497 & 6.749 & 10.132 & 15.203\\ -0.001 & 0.002 & 0.009 & 0.004 & 0.006 \end{array}\right]$$

The parameters used for this example are *μ* = 0. 1, = 1.5, and δ = 1, giving an unstable system. The random disturbances for the input are generated with zero mean and gaussian distributed with a variance of 0.01. Six snapshots of data are used for the computation. Using these data matrices, a restricted Koopman operator can be constructed. The solution using these data matrices is

(4.5) $$G=\left[\begin{array}{l} G_{11}\;G_{12}\\ G_{21}\;G_{22} \end{array}\right]\approx \left[\begin{array}{ccc} \left[\begin{array}{cc} 0.1 & 0\\ 0 & 1.5 \end{array}\right] & \left[\begin{array}{l} 0\\ 1 \end{array}\right] & \;\\ {}[-.0005 & 0.001] & \left[-0.127\right] \end{array}\right]$$

The underlying system (4.2) is reconstructed with the random disturbances for inputs. Note that G_11_ and G_12_ are accurately identified from the data. The restricted Koopman operator also attempts to fit G_21_ and G_22_ as a propagator on the random inputs, which will not be accurate by construction.

If the input is generated from a controller with state-feedback, then the data in the last row becomes correlated with the second row. For example, we define a state-dependent controller *u* = *−Kx*_2_, where *𝓚* = 1. In order to disambiguate the control from the observable *y*_2_, a small disturbance is added to the input *y*_3_ in the data matrix **Y**. This provides the following approximate restricted Koopman operator:

(4.6) $$G=\left[\begin{array}{l} G_{11}\;G_{12}\\ G_{21}\;G_{22} \end{array}\right]\approx \left[\underset{\left[\begin{array}{cc} 0 & -1.5 \end{array}\right]}{\left[\begin{array}{cc} 0.1 & 0\\ 0 & 1.5 \end{array}\right]}\;\underset{\left[-1\right]}{\left[\begin{array}{l} 0\\ 1 \end{array}\right]}\right]\;,$$

where the dynamics on the controller now mimic the actual dynamics of *x*_2_. In this example, the restricted Koopman operator recovers the unstable underlying dynamics and discovers that the inputs are being generated by a controller that is dependent on *x*_2_. This example motivates our generalization of Koopman theory. The Koopman operator, without consideration of the external inputs or control, would only be able to recover stable dynamics, thus fundamentally mischaracterizing the underlying system.

Consider the final input type: the input has dynamics. The dynamics are not state dependent, for example $\dot{u}=-ru$ with *r* = 0.01 and *u*(0) = 1. We collect the data and find a restricted Koopman operator:

(4.7) $$G=\left[\begin{array}{l} G_{11}\;G_{12}\\ G_{21}\;G_{22} \end{array}\right]\approx \left[\underset{\left[\begin{array}{cc} 0 & 0 \end{array}\right]}{\left[\begin{array}{cc} 0.1 & 0\\ 0 & 1.5 \end{array}\right]}\;\underset{\left[0.99\right]}{\left[\begin{array}{l} 0\\ 1 \end{array}\right]}\right]\;,$$

The KIC architecture not only discovers the underlying dynamics of **x** and the impact of *u*, but also finds the dynamics on *u* . This perspective could be beneficial when considering multiscale modeling in climate science or epidemiology.

The restricted KIC operator can be recovered from the data despite the unstable eigenvalue and various types of inputs. Note that both operators **A** and **B** are recovered from the underlying dynamical system (4.2). The left Koopman modes are

(4.8) $$q=\left[\begin{array}{cc} 1 & 0\\ 0 & 1 \end{array}\right],$$

where these Koopman modes can be used to construct the eigenfunctions ψ*_j_*, described in section 3.4.2. A similar procedure can be utilized to find the right Koopman modes v*_j_* and eigenfunctions *ϕ_j_*.

### 4.2. Example 2—Nonlinear system with inputs

We demonstrate how KIC can be used to solve a nonlinear example with inputs. We utilize the modified KIC definition where u*_k_*_+1_ = 0. Consider the following nonlinear dynamical system from [48] and [4], modified to include an input *u*:

(4.9) $$\left[\begin{array}{c} {\dot{x}}_{1}\\ {\dot{x}}_{2} \end{array}\right]=\left[\begin{array}{c} \mu x_{1}\\ \lambda \left(x_{2}-x_{1}^{2}\right)+\delta u \end{array}\right]$$

where λ = 0.5, *μ* = 2, and γ = 2. We use this example to investigate the e ect of inputs and control on the nonlinear system. The observables are carefully chosen, as in [4], to investigate this dynamical system:

(4.10) $$\left[\begin{array}{l} y_{1}\\ y_{2}\\ y_{3}\\ y_{4} \end{array}\right]=\left[\begin{array}{c} x_{1}\\ x_{2}\\ x_{1}^{2}\\ u \end{array}\right],\;\left[\begin{array}{c} {\dot{y}}_{1}\\ {\dot{y}}_{2}\\ {\dot{y}}_{3} \end{array}\right]=\left[\begin{array}{ccc} \mu & 0 & 0\\ 0 & \lambda & -\lambda \\ 0 & 0 & 2\mu \end{array}\right]\left[\begin{array}{c} y_{1}\\ y_{2}\\ y_{3} \end{array}\right]+\left[\begin{array}{c} 0\\ \delta \\ 0 \end{array}\right]y_{4}$$

where the nonlinear function $y3=x_{1}^{2}$ has a convenient derivative which allows $\dot{y3}$ to be represented by the other observables; see [4] for more information about closure and Koopman invariant subspaces. We can transform the problem to include the inputs:

(4.11) $$\left[\begin{array}{l} {\dot{y}}_{1}\\ {\dot{y}}_{2}\\ {\dot{y}}_{3} \end{array}\right]=\left[\begin{array}{l} \mu \;0\;0\;0\\ 0\;\lambda \;-\lambda \;\delta \\ 0\;0\;2\mu \;0 \end{array}\right]\left[\begin{array}{l} y_{1}\\ y_{2}\\ y_{3}\\ y_{4} \end{array}\right]$$

Now, we collect measurement data in terms of the observables. In this numerical example, we used 15 iterations with an initial condition of ${\left[x_{1}\;x_{2}\right]}^{T}={\left[5\;2\right]}^{T}$. The restricted Koopman operator on these observables can be reconstructed:

(4.12) $$G=\left[\begin{array}{l} G_{11}\;G_{12} \end{array}\right]\approx \left[\begin{array}{cccc} 2 & 0 & 0 & 0\\ 0 & 0.5 & -0.5 & 2\\ 0 & 0 & 4 & 0 \end{array}\right]$$

The left Koopman modes can be constructed similarly to [4] and as described in (2.13). These Koopman modes **q***_j_* can then be used to construct eigenfunctions ψ*_j_* (**x**) = *〈*x, q*_j_〉*. These eigenfunctions span the subspace of observables in the output space. The right Koopman modes and eigenfunctions can also be computed as described by (2.13). The right eigenfunctions span the domain space. Despite the nonlinear dynamical system, the KIC perspective constructs a linear dynamical system on the measurements that can be used for prediction and control.

### 4.3. Example 3—A biologically inspired nonlinear example

**Figure 3.**
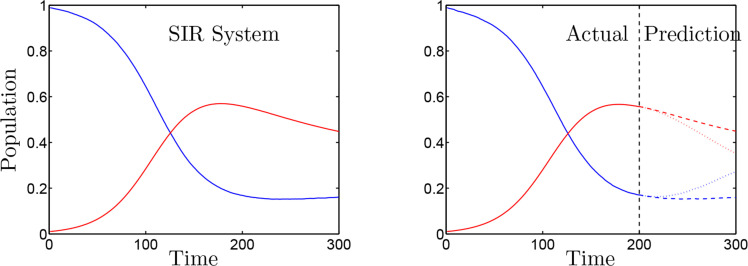
Left panel: SIR dynamics with a 1*% seeding of infection at time zero. Right panel: The same SIR dynamics left of the dark dashed line. To the right of the line, the dotted line indicates the KIC prediction with only the linear observables* (*S, I, R*) as the output. The dashed line indicates the prediction if the measurements (*S, I, R, SI*) are used for the output.

We investigate KIC on the classic SIR model. This example contains a nonlinearity which is fundamentally di erent from Example 2. The nonlinearity does not have the same closure property. Consider one version of the SIR models with inputs (represented by the vaccination of susceptible individuals):

(4.13) $$\left[\begin{array}{c} \dot{S}\\ \dot{I}\\ \dot{R} \end{array}\right]=\left[\begin{array}{c} -\beta SI\;+\;\nu \left(S+I+R\right)-\mu S-\mathrm{Vacc}\\ \beta SI-\gamma I-\mu I\\ \gamma I-\mu R+\mathrm{Vacc} \end{array}\right],$$

where β = 10 is an infectious parameter, = 1 is a birthrate parameter depending on the total population of the community *S* + *I* + *R* = 1, *μ* = 1 is the death rate, = 1 is a recovery rate from infection, and Vacc is a rate of vaccination. The left panel of Figure 3 shows the SIR system dynamics with seeding a 1% infection at time zero and adding a small random amount of vaccination at each time step. The nonlinearity in this example, (*SI*), is a mixed state quadratic nonlinearity. We transform this continuous nonlinear dynamical system into a discrete linear dynamical system with a simple forward-Euler scheme, augment the domain space to include the nonlinearity *SI*, and include inputs. The following are the observables for the domain and output variables:

(4.14a) $$\text{Domain}\;{\left[y_{1}\;y_{2}\;y_{3}\;y_{4}\;y_{5}\right]}^{T}={\left[S\;I\;R\;SI\;\text{Vacc}\;\right]}^{T}\;$$

(4.14b) $$\text{Output:}\;{\left[y_{1}\;y_{2}\;y_{3}\right]}^{T}={\left[S\;I\;R\right]}^{T}.\;$$

Using these domain and output observables, the system can be rewritten:

(14.15a) $${\left[\begin{array}{c} y_{1}\\ y_{2}\\ y_{3} \end{array}\right]}_{k+1}=\left[\begin{array}{cccc} 1/\left(∆t\right)-\mu +\nu & v & \nu & -\beta \\ 0 & 1/\left(∆t\right)-\mu -\gamma & 0 & \beta \\ 0 & \gamma & 1/\left(∆t\right)-\mu & 0 \end{array}\begin{array}{c} -1\\ 0\\ 1 \end{array}\right]{\left[\begin{array}{c} y_{1}\\ y_{2}\\ y_{3}\\ y_{4}\\ y_{5} \end{array}\right]}_{k}\;$$

(14.15b) Z=KY,

where **K** is the KIC operator, **Z** is the data in the output observables, and **Y** is the data in the input observables. Choosing a tailored set of domain and output observables introduces a practical di culty in the implementation of a completely model-free methodology. For example, the inclusion of the nonlinear term (*SI*) with (*S, I, R*) to the domain observables allows for a well-characterized Koopman operator from the training data. Further, this Koopman operator can be used for prediction and comparison to an out-of-sample dataset. The right panel of Figure 3 illustrates the training data, in solid lines, versus the out-of-sample prediction, in dashed lines. The out-of-sample prediction matches the actual system seen in the left panel of Figure 3. In order to construct the future prediction, the nonlinear observable (*SI*)*_k_* is constructed from *S_k_* and *I_k_* for each application of the Koopman operator.

If (*SI*) is also included in the output observables, then the approximate Koopman operator will have one more row in (4.15a). The goodness-of-fit for this new row will be poor due to the lack of higher order terms required to characterize $\frac{d}{dt}\left(SI\right)$, given that the derivative does not lend itself to a closed form. The derivative property $\frac{d}{dt}\left(SI\right)=\dot{S}I+S\dot{I}$ introduces the need for even more nonlinearities to characterize the output observables, thus increasing the required number of augmented observables. However, the goodness-of-fit for the rows describing the evolution of *S*, *I*, and *R* will still be quite good for the in-sample training data. The practical limitation arises in the out-of-sample prediction. Since the evolution of (*SI*) is not well-described without adding more observables, the use of (*SI*)*_k_* to predict *S_k_*_+1_ will quickly cause the prediction to diverge from the real solution. This can be seen by the dotted line in the right panel of Figure 3.

Choosing the correct observables is of paramount importance. A similar sentiment is expressed in [50], but without considering separate domain and output spaces. The inclusion of new observables for either the domain or the output side requires examination of goodness-of-fit metrics on the training data as well as out-of-sample prediction tests. Further, recent work has shown a statistical framework for determining which nonlinearities to include by sparsely choosing from a large library of possible dynamical terms [6].

## 5. Discussion

A wealth of modern applications are nonlinear and high-dimensional including distribution systems, internet tra c, and vaccination of human populations in the developing world. The need to develop quantitative and automatic methods to characterize and control these systems is of paramount importance to solving these large-scale problems. In order to construct e ective controllers, the complex system has to be well-understood. In the case that we do not have well-established, physics-based governing equations, equation-free methods can help characterize these systems and o er insight into their control.

Koopman operator theory and DMD o er a data-driven method to characterizing complex systems [31, 42]. These methods are strongly grounded in the analysis of nonlinear systems and have been successfully applied in a number of fields such as fluid dynamics [31, 46, 45, 2], epidemiology [37], video processing [14], and neuroscience [3]. Further, this architecture has allowed for the incorporation of recent innovations from compressive sensing providing insight into optimally measuring a system [21, 48, 7]. Generalizing Koopman for input-output systems allows for a broader set of systems to be considered. KIC is well-connected to DMDc, which is already having an impact analyzing input-output characteristics for systems with linear observables [36, 10].

Theoretical innovations such as KIC will play an ever-increasing role in the control of complex systems. Specifically, the characterization of the domain and output spaces of the KIC operator can provide insight into the design of nonlinear estimators and controllers solely from data. We also expect these ideas to open up new theoretical research directions, such as investigating the implications of KIC for measure-preserving flows and the design of novel control profiles for rapid and e cient nonlinear system identification. A practical challenge, though, facing the widespread deployment of Koopman operator theory is choosing a set of nonlinear observables that allow for the construction of a finite-dimensional, linear operator that actually represents the nonlinear evolution of the system. Despite this current limitation, we believe KIC and DMDc are well poised to be integrated into a diverse set of engineering and science applications. KIC is positioned to have a significant impact in the analysis and control of large-scale, complex, input-output systems.
